# Probing the Frontostriatal Loops Involved in Executive and Limbic Processing via Interleaved TMS and Functional MRI at Two Prefrontal Locations: A Pilot Study

**DOI:** 10.1371/journal.pone.0067917

**Published:** 2013-07-09

**Authors:** Colleen A. Hanlon, Melanie Canterberry, Joseph J. Taylor, William DeVries, Xingbao Li, Truman R. Brown, Mark S. George

**Affiliations:** 1 Department of Psychiatry, Medical University of South Carolina, Charleston, South Carolina, United States of America; 2 Center for Biomedical Imaging, Medical University of South Carolina, Charleston, South Carolina, United States of America; 3 Department of Radiology, Medical University of South Carolina, Charleston, South Carolina, United States of America; 4 Ralph H. Johnson Department of Veterans Affairs Medical Center, Charleston, South Carolina, United States of America; Yale University School of Medicine, United States of America

## Abstract

**Background:**

The prefrontal cortex (PFC) is an anatomically and functionally heterogeneous area which influences cognitive and limbic processing through connectivity to subcortical targets. As proposed by Alexander et al. (1986) the lateral and medial aspects of the PFC project to distinct areas of the striatum in parallel but functionally distinct circuits. The purpose of this preliminary study was to determine if we could differentially and consistently activate these lateral and medial cortical-subcortical circuits involved in executive and limbic processing though interleaved transcranial magnetic stimulation (TMS) in the MR environment.

**Methods:**

Seventeen healthy individuals received interleaved TMS-BOLD imaging with the coil positioned over the dorsolateral (EEG: F3) and ventromedial PFC (EEG: FP1). BOLD signal change was calculated in the areas directly stimulated by the coil and in subcortical regions with afferent and efferent connectivity to the TMS target areas. Additionally, five individuals were tested on two occasions to determine test-retest reliability.

**Results:**

Region of interest analysis revealed that TMS at both prefrontal sites led to significant BOLD signal increases in the cortex under the coil, in the striatum, and the thalamus, but not in the visual cortex (negative control region). There was a significantly larger BOLD signal change in the caudate following medial PFC TMS, relative to lateral TMS. The hippocampus in contrast was significantly more activated by lateral TMS. Post-hoc voxel-based analysis revealed that within the caudate the location of peak activity was in the ventral caudate following medial TMS and the dorsal caudate following lateral TMS. Test-retest reliability data revealed consistent BOLD responses to TMS within each individual but a large variation between individuals.

**Conclusion:**

These data demonstrate that, through an optimized TMS/BOLD sequence over two unique prefrontal targets, it is possible to selectively interrogate the patency of these established cortical-subcortical networks in healthy individuals, and potentially patient populations.

## Introduction

Although there are many ways to parse the primate prefrontal cortex (PFC) anatomically and functionally, one of the most frequently cited divisions include the dorsal-lateral (DLPFC) and the ventral-medial portions (MPFC) of the PFC [1]. Alexander and colleagues (1986) proposed that these regions have parallel architecture, but remain functionally and anatomically distinct as they project to the striatum and then onto the thalamus. Additionally, these parallel functionally segregated pathways from the DLPFC and the MPFC have unique roles in shaping executive and limbic processing [2], [3].

The DLPFC and the MPFC both receive afferent information from the medial-dorsal thalamus and send efferent fibers to subcortical areas. The primary subcortical targets of the DLPFC in primates include the dorsal striatum and hippocampus. These areas are typically activated during executive processing tasks including logical decision making [4] and working memory [5], [6], [7]. The primary subcortical targets of the ventral-medial prefrontal cortex include the ventral striatum [8] and amygdala [9]. These areas are typically activated during by tasks with a high level of affective/limbic valence [10] including reward based learning [11], anticipation of monetary rewards [12], and humor [13], [14].

There is a well-established association between hyperactive medial prefrontal cortex circuitry and psychiatric disease including psychopathy [15], [16], eating disorders [17], and substance dependence [18], [19], [20]. As these lateral and medial prefrontal circuits likely interact to balance cortical control of affective responses, several imaging studies have attempted to differentiate dorsolateral from ventromedial activity [21], [22]. The correlative nature of traditional functional connectivity analysis however, hinders conclusions regarding causal links between tasks and ensuing distributions of activity.

Transcranial magnetic stimulation (TMS) is a non-invasive brain stimulation technique which, when performed in the MR environment, may enable us to differentially probe activity in these lateral and medial prefrontal circuits which are engaged in executive and limbic processing. Extensive motor cortex literature has established that single-pulse TMS leads to elevated activity in the cortex directly affected by the induced electrical field, as well as cortical and subcortical areas monosynaptically connected to the site [23], [24], [25], [26], [27], [28], [29]. Additionally, subcortical dopamine binding is elevated following TMS to the DLPFC [30], [31]. The literature on TMS-BOLD in the prefrontal cortex however, is limited to a few studies of the DLPFC [32], [33], [34]. Presently there are no published reports of TMS-BOLD imaging to the medial prefrontal cortex.

The primary purpose of this pilot investigation was to determine if, through the use of an optimized interleaved TMS-BOLD sequence with two distinct prefrontal cortical targets, we could differentially activate the DLPFC and the MPFC as well as downstream subcortical targets. Given that this is the first study to acquire functional imaging data following TMS to the MPFC as well as the DLPFC in the MRI scanner we started with a relatively small cohort of 17 healthy individuals with no history of neurologic of psychiatric disease. In addition to assessing the feasibility and safety of acquiring robust BOLD signal from both the MPFC and the DLPFC in a single session, we also sought to determine whether stimulation in these regions was associated with differential activation of subcortical targets. Finally, test-retest reliability of TMS-induced BOLD signal change was acquired in 5 individuals. These studies were all performed with the intent that this may be extended to assessment of treatment interventions in psychiatric populations in which the functional integrity of these executive and limbic circuits may be compromised.

## Materials and Methods

### Participants and Ethics Statement

Seventeen healthy individuals were recruited from the community and provided a written and oral informed consent consistent with the principles expressed in the Declaration of Helsinki. The Medical University of South Carolina Institutional Review Board approved this study. Participants were medically and neurologically healthy (age: 21–45) with no history of brain injury, loss of consciousness for more than 15 seconds nor a history of psychiatric disease.

### Motor Threshold

Participants were brought to the imaging center where resting motor threshold (RMT) was determined. TMS was applied using a Magstim SuperRapid stimulator which generates biphasic electrical pulses (250 µs). The stimulator was located outside of the scanning room and the cable that is typically attached to the TMS coil was attached to an RF filter. The pulses were delivered through a 8meter cable which first attached to the bottom of an RF filter, and then passed through the waveguide into the MR scanning room where it was led through the bore of the MRI and terminated in a custom nonferromagnetic figure-of-eight TMS coil [32], [33], [35], [36].

### Coil Positioning

**Figure 1:**
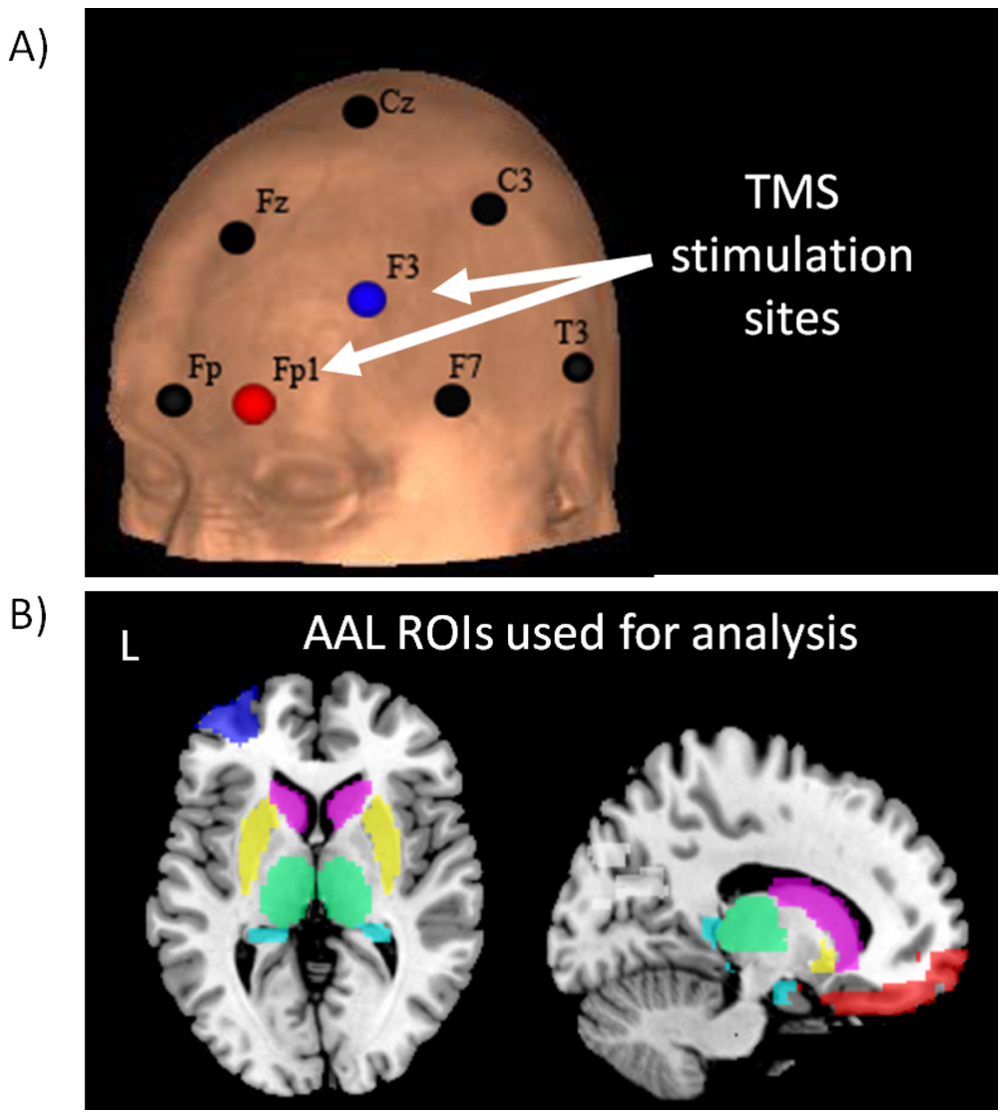
TMS stimulation sites and anatomical regions of interest. TMS was applied at two locations the DLPFC (F3, blue circle) and the MPFC (FP1, red circle), shown here on a standardized anatomical image (black circles = standard positions in the 10–20 system) (A). Regions of interest for analysis were defined apriori using standardized regions from the Automated Anatomical Labeling atlas (AAL) and included the middle frontal gyrus (blue), superior and middle orbital gyri (red)), caudate (pink), putamen (yellow), amygdale (orange), hippocampus (teal), and thalamus (green) (B). Additionally the cuneus was chosen as a control region (dark gray).

**Figure 2:**
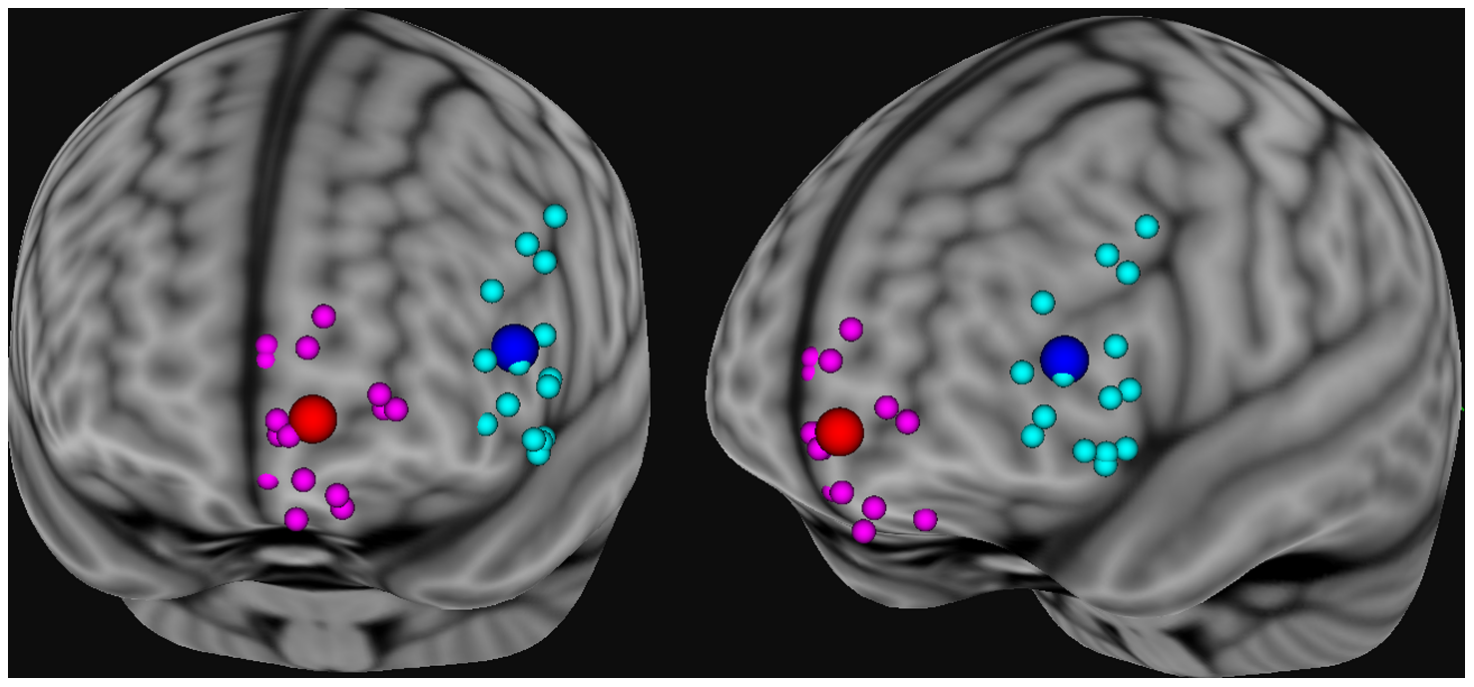
The location of the peak BOLD response for each individual following TMS pulses to the F3 location (blue spheres) and to the FP1 location (red spheres). The locations of the peak BOLD response for each individual have be normalized to standard space and projected to the surface of standard cortical mask such that all points are visible in a common space. The mean location of peak activity in the cluster beneath the coil is shown (larger blue and red spheres).

Participants were positioned supine on the scanner bed and the TMS coil was mounted in the MR head coil with a custom TMS coil holder adjustable in 6 directions (X,Y, Z, pitch, yaw, roll) [35]. The standardized international 10–20 system for EEG electrode placement was used as the basis for positioning the TMS coil as it accounts for variability in participant skull size and is consistently used in clinical TMS applications (**Figure 1A**). Although there is some debate regarding the selection of F3 or F5 is the optimal location for targeting the DLPFC [37], in this study we chose F3 based on compatibility with prior TMS-BOLD imaging papers [32], [33], [38]. This resulted in a site of stimulation that was just dorsal to the midaxial plane and consequently activation largely in BA 46 and the lateral aspects of BA9 (see Figure 2, blue spheres). For MPFC stimulation FP1 (frontal pole, left side) was the chosen location for TMS. Although this is shifted to the left ventral medial cortex, TMS stimulation at Fz (midline) would likely have been too dorsal to effect limbic circuitry and may have been associated with an attenuated response due to the high amounts of cerebrospinal fluid between the skull and cortex in that location. Consequently, while participants lay supine on the bed the position of the TMS coil was aligned to the location of F3 (Position 1: left DLPFC; Figure 1A: red sphere) and to FP1 (Position 2: MPFC; Figure 1A: blue sphere). A fiudicial was affixed to the center of the TMS coil for offline verification of position.

### Image Acquisition

This study was performed on a Siemans 3T TIM trio scanner (Siemens, Erlangen, Germany) with a twelve-channel RAPID Biomedical (Rimpar, Germany) head coil. High-resolution T_1_-weighted anatomical images were acquired for each participant (TR = 1750 ms, TE = 4 ms, voxel dimensions 1.0×1.0×1.0 mm, 160 slices).

Following anatomical image acquisition and alignment, participants received 2 interleaved TMS-BOLD imaging runs with the coil positioned over the F3 or FP1. After each run the bed of the scanner was retracted from the bore of the magnet and the coil was moved to the other location such that all participants received a TMS/BOLD imaging run with stimulation at each of the two locations. Extensive pilot work and prior studies in our laboratory were used to determine that TMS-induced artifacts in the EPI data were minimized using a sparse acquisition technique in which the biphasic TMS pulse (250 µs) was applied during a 100 ms gap between 2 volumes [26], [39] Accordingly, each interleaved TMS-BOLD run consisted of 6 TMS pulses (100% motor threshold), applied during a 100 ms gap between EPI image acquisition (flip = 90degrees, TR = 2.52 sec, TE = 0.023 s, FOV = 230 mm, voxel size = 3×3×3). [Note: Although this is a very conservative number of TMS pulses and likely lowers our potential signal to noise ratio, this cautious design was chosen for our preliminary investigation because there was no precedent for stimulation of the MPFC in the MRI scanner]. The interpulse interval was 10.18 s. Forty two volumes of data were acquired for each run with the first 6 discarded to ensure magnetic field homogeneity. After the first functional run, the bed of the MRI scanner was retracted from the bore such that the TMS coil could be moved to the second position. Low resolution T_1_-weighted anatomical images were acquired again for alignment and offline verification of coil position, before commencing the second TMS-BOLD imaging run. For quality control purposes each EPI volume was inspected for TMS induced signal artifacts both before and after temporal realignment. Consistent with prior studies from our group this timing produced artifact-free EPI images for 100% of the participants [34].

### Functional MRI Data Analysis

Spatial preprocessing was performed with standard parametric mapping techniques (SPM8, London, UK) in MATLAB 7.0 (Mathworks, Natick, MA) and custom scripts. The data were corrected for acquisition time (slice timing), realigned to the first volume (motion correction), normalized into a standardized neuroanatomical space (Montreal Neurological Institute (MNI) brain template), and smoothed using a Gaussian kernel of 8 mm for the group analysis to reduce the variance due to anatomical variability. Analyses of time data series were performed individually modeling the TMS pulse as an event convolved with the canonical hemodynamic response function. Statistical contrast maps were made for each individual comparing brain activity associated with performing the task to that during periods of rest. These data were modeled across all participants in order to obtain a voxel-based representation of brain areas being used to perform the task for each group. These first-level contrast maps were used to determine the location of peak activity for each individual following the TMS pulses at both the medial and lateral sites (**see** **Figure 2**).

### Regions of Interest

An anatomically-defined region of interest (ROI) analysis was used to examine BOLD signal changes in the brain regions beneath the coil as well as in subcortical projection regions. The ROIs were defined based on the automated anatomical labeling system (AAL) (**Figure 1B**). The MPFC ROI included the left superior orbital and middle orbital regions. These ventral medial areas were all inferior to the anterior commissure (z <0). The DLPFC ROI included the left middle frontal gyrus, which cointains BA 46 and lateral aspects of BA9 and BA10. Note: Although these ROI are much larger than the 2 cm^2^ area likely to be maximally effected by a TMS pulse at the motor threshold, many other interleaved TMS-BOLD imaging studies have demonstrated an increase in BOLD signal in areas that extend several centimeters from the site of stimulation [25], [29], [40]. Consequently, while using a large ROI likely decreases our signal to noise ratio, it also enables us to use a standardized atlas which minimizes bias and likely enhances the ability of others to replicate the results. To assess the impact of TMS on other elements of the frontostriatal loops, regions with known monosynaptic efferent (caudate and putamen) and afferent (thalamus) connectivity with the DLPFC and MPFC were also chosen from the AAL atlas. Finally, the hippocampus and amygdale were also included in the analysis as they both project to the PFC, and are critical regulators of memory and emotion processing. The primary visual cortex was chosen as a negative control region. This was done as the visual cortex (AAL: cuneus) is not likely to be activated by either stimulation site.

The average timecourse from each ROI was extracted using from the preprocessed time series during both the F3 and the FP1 stimulation for each individual. Signal drift between runs was addressed through global scaling (MarsBaR 0.41) in accordance with similar studies by our group [41], [42]. The average BOLD signal within each ROI was extracted for the full timecourse of the task. The magnitude of the TMS-related activity was measured by calculating the maximum percent signal change (percent signal change) between the prestimulus baseline and the 10.18 s after the TMS pulse. The average peak signal change was compiled across individuals and compared using analysis of variance (ANOVA, TMS location x ROI) with posthoc Student t-tests in the event of an interaction. These analyses were done explicitly for 1) the cortical ROIs under the coil in which percent signal change is likely due to a direct effect of TMS induced neural activity, and 2) the subcortical ROIs likely to be indirectly effected by the TMS pulse along with one negative control ROI.

### Posthoc Voxel Based Analysis

The primary purpose of this preliminary investigation was to determine whether we could differentially activate the cortical and the subcortical elements of well-established frontostriatal circuits via TMS. This was done using apriori defined anatomical regions of interest using an established atlas, which were significantly larger that the discrete populations of neurons we expect to be activated by the TMS pulse, either directly or secondarily. To further extend our findings to other regions of the brain which may have been affected by the TMS pulse, we performed a whole brain posthoc voxel-based analysis comparing MPFC stimulation to DLPFC stimulation. For this voxel-based analysis TMS pulses were modeled as discrete events and temporally convolved with the canonical hemodynamic response function. Statistical contrast maps of these events were made for each individual (voxel-level, p<0.005, cluster minimum = 25) and compared between sites (cluster correction, p<0.05, cluster minimum = 25).

### Test-Retest Reliability

The first five participants completed a second visit with the same TMS stimulation strength (two women, 27–49 yrs old, one with prior exposure to BOLD-TMS). The percent signal change in the cortical areas near the site of stimulation (MPFC, left DLPFC) were compared within and between individuals for each visit.

## Results

### Spatial Topography of Peak BOLD Signal

As described above, the EEG coordinate system was used as a guide for TMS coil placement. Given individual variability in the shape of the skull, amount of cerebrospinal fluid between the skull and cortex, and in gyral folding, there is likely to be some variability in the location of peak BOLD signal subsequent to TMS. In order to determine and validate the location of the brain directly stimulated by the TMS pulse, for each individual we isolated the coordinates of the peak voxel in a cluster activated by the TMS pulse. This was done for both runs and these locations were projected to the surface of the curvilinear brain mask such that all points would be visible (**Figure 2**). The mean projected location of stimulation for the DLPFC run (EEG:F3) was located in BA 46 (x,y,z: −49, 39, 13) whereas the mean location for the MPFC run (EEG:FP1) was located in BA 10 (x,y,z: −13, 67, −1). The distribution of these points did not overlap and the average distance between the mean of the MPFC and DLPFC was 39.19 mm. and the average distance to the mean was 9.22 mm.

### Region of Interest

**Figure 3:**
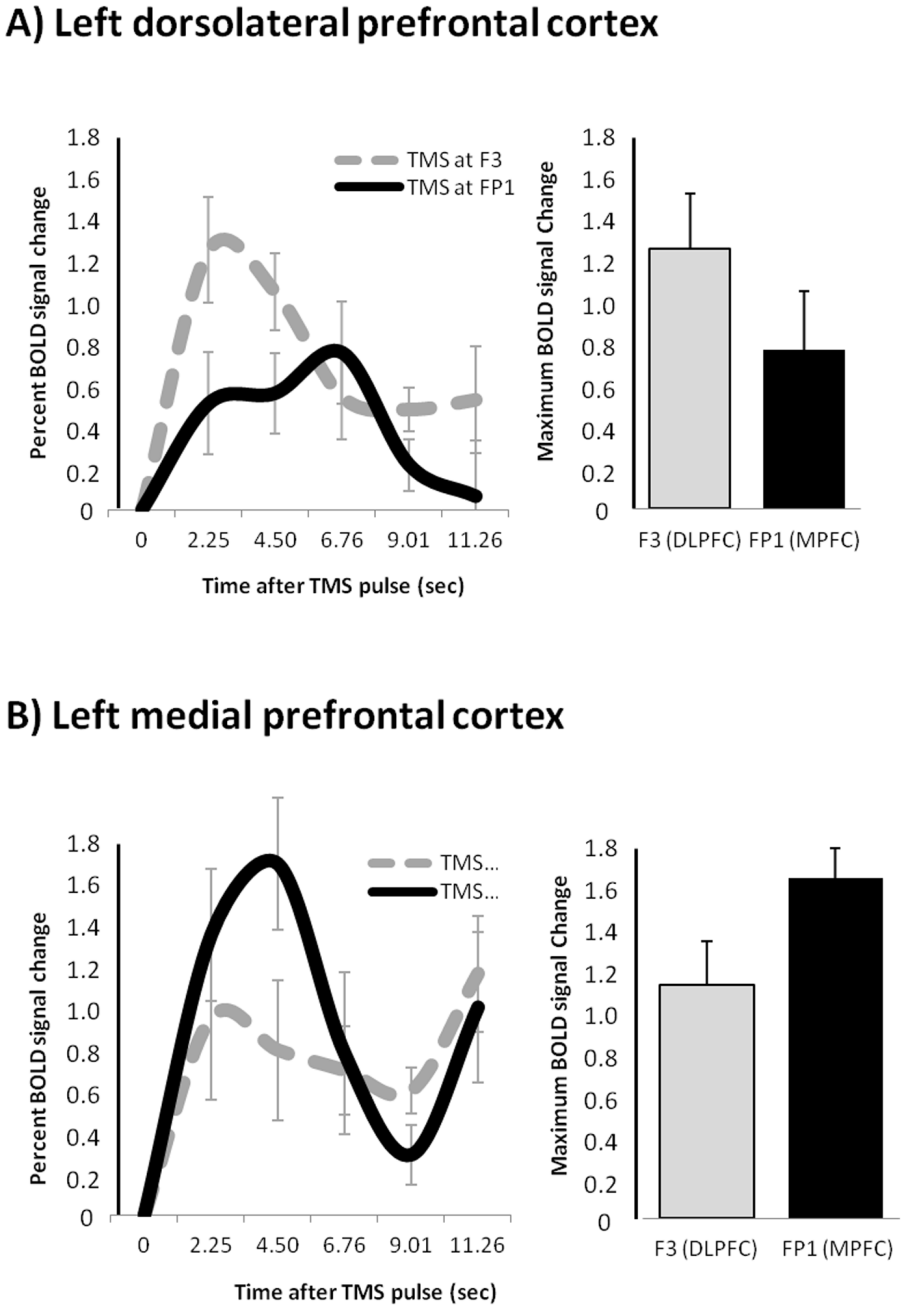
Hemodynamic response in the left DLPFC and MPFC following stimulation at F3 and FP1.

Both lateral and medial TMS targets were associated with an elevation in BOLD signal in cortex adjacent to the site of stimulation (**Figure 3**). Percent signal change in the DLPFC was significantly greater for the lateral (F3) TMS target (mean ±sd; 0.120±0.003) than for the medial (FP1) target (0.07±0.003; p<0.001, Figure 3A). Percent signal change in the MPFC, correspondingly, was significantly greater for the medial (FP1) TMS target (0.149±0.003) than for the lateral (F3) target (0.102±0.003; p<0.001, Figure 3B).

**Figure 4:**
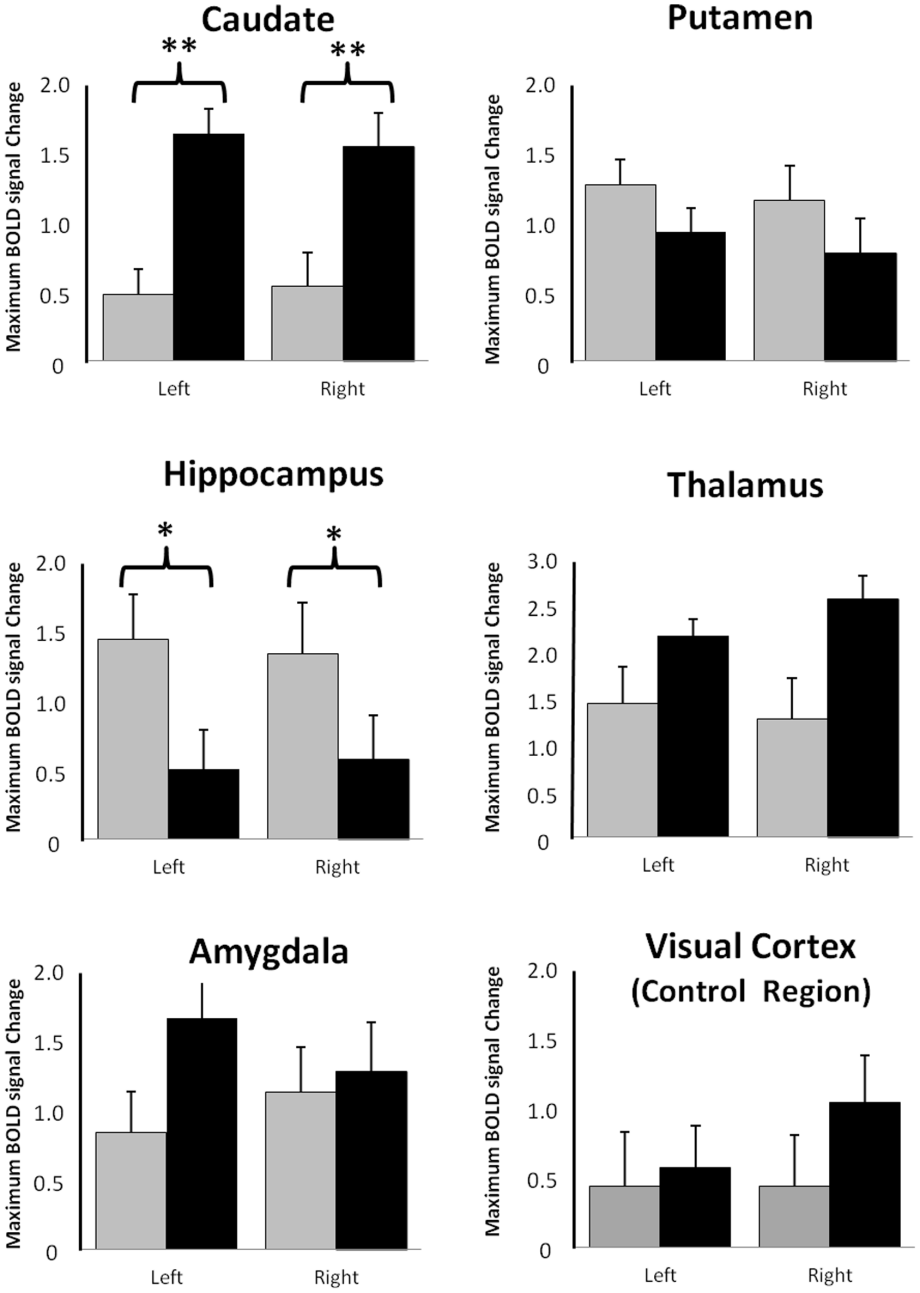
Percent BOLD signal change in subcortical brain regions monosynaptically connected to the left DLPFC or MPFC following stimulation at the DLPFC site (F3) (gray) and the MPFC site ((FP1) (black). The left and right portions of each ROI are displayed. *p<0.005 **p<0.005.

There was a significant interaction between the site of prefrontal TMS stimulation and the BOLD response in the 6 subcortical brain regions (F(5,176) = 7.9, p<0.0001). (**Figure 4**) The percent signal change in the caudate was significantly greater following MPFC stimulation than DLPFC stimulation (F(1,16) = 12.2, p = 0.0030), no difference between hemispheres). The percent signal change in the putamen however, was not differentially affected by lateral versus medial PFC TMS. The hippocampus was significantly more active following DLPFC TMS (F(1,16) = 5.3, p = 0.0351), no difference between hemispheres). The amygdala was not differentially activated by the location of TMS, and percent signal change in the visual cortex (control region) was not significantly different from baseline overall. The largest percent signal change of all regions investigated was in the thalamus. Although there was no effect of TMS site on thalamic response to stimulation, the percent signal change in the thalamus was higher following medial versus lateral stimulation in 13 of the 17 participants.

### Post-hoc Voxel Based Analysis

**Figure 5:**
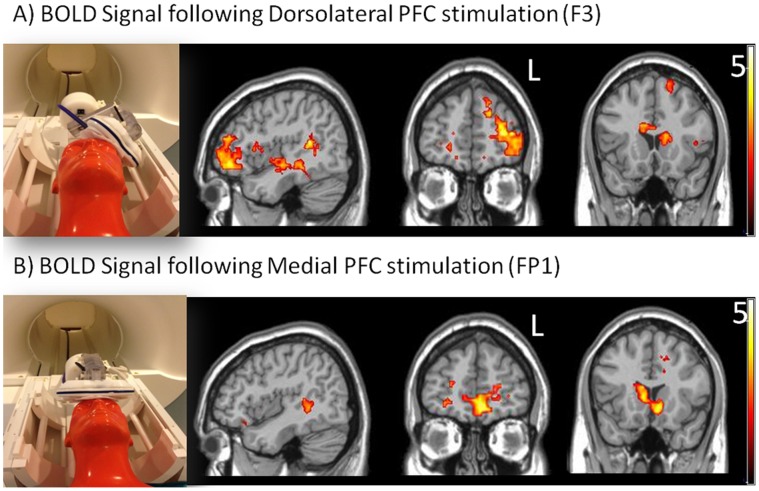
Spatial distribution of regions significantly activated by single-pulse TMS to the left DLPFC (A) and the MPFC (B). A model of the placement of the TMS coil in the MR environment is shown in the far left panel. Statistical contrast maps of change in BOLD signal following a TMS pulse are shown in the panels to the right (t values displayed on colorbar, maximum 5).

Left DLPFC stimulation (F3) was associated with significantly elevated activity in the left middle frontal gyrus (Brodmann Area (BA) 10, xyz (−27,47,10), maximum t-value: 4.7, k = 655, p<0.001 cluster level), right superior frontal gyrus (BA 6, (24,62,16), t-value: 4.29, cluster size: 245, p = 0.001), left superior temporal sulcus (BA 41,42, (−33, −40, 4), t-value: 5.16, 697, p<0.001), dorsal caudate ((24, −22, 28), t-value:6.43, cluster size:220, p = 0.001). (**Figure 5A**).

Medial PFC stimulation (FP1) was associated with elevated activity in bilateral orbitofrontal cortex (BA 11 (6,23, −23), t-value: 5.86, cluster size: 191, p = 0.003), bilateral anterior cingulate cortex (BA 32 (18,44, −11), t-value: 4.81, cluster size: 287, p<0.001), right ventral caudate ((30, −40, 10), t-value:4.62, cluster size: 153, p = 0.006), bilateral dorsal caudate ((−9, 14, 4), t-value: 4.37, cluster size: 256, p = 0.001). (**Figure 5B**) Furthermore, MPFC stimulation was associated with a decrease in BOLD signal (relative to resting baseline) in the bilateral superior frontal gyrus (BA8, (3,35,49), t-value: 4.71, cluster size: 293, p<0.001).

Relative to MPFC stimulation, left DLPFC stimulation was associated with significantly more activity in the left insula/inferior frontal cortex (BA 44,45,6 (−48,14,3), t-value: 6.30, cluster size: 301, p = 0.004) and right lateral PFC (BA9 (48, −58, 55), t-value: 5.44, cluster size: 61, p = 0.005). In contrast, MPFC stimulation was associated with significantly more activity in the ventral caudate ((−12, −11, −2), t-value: 3.85, cluster size: 144, p = 0.008), cingulate cortex (BA 32 (−3, 53, −11), t-value: 3.92, cluster size: 76, p = 0.04), and precuneus (BA7 (−1, −10, 46), t-value: 4.58, cluster size: 270, p = 0.001) relative to DLPFC stimulation.

### Test-Retest Reliability

**Figure 6:**
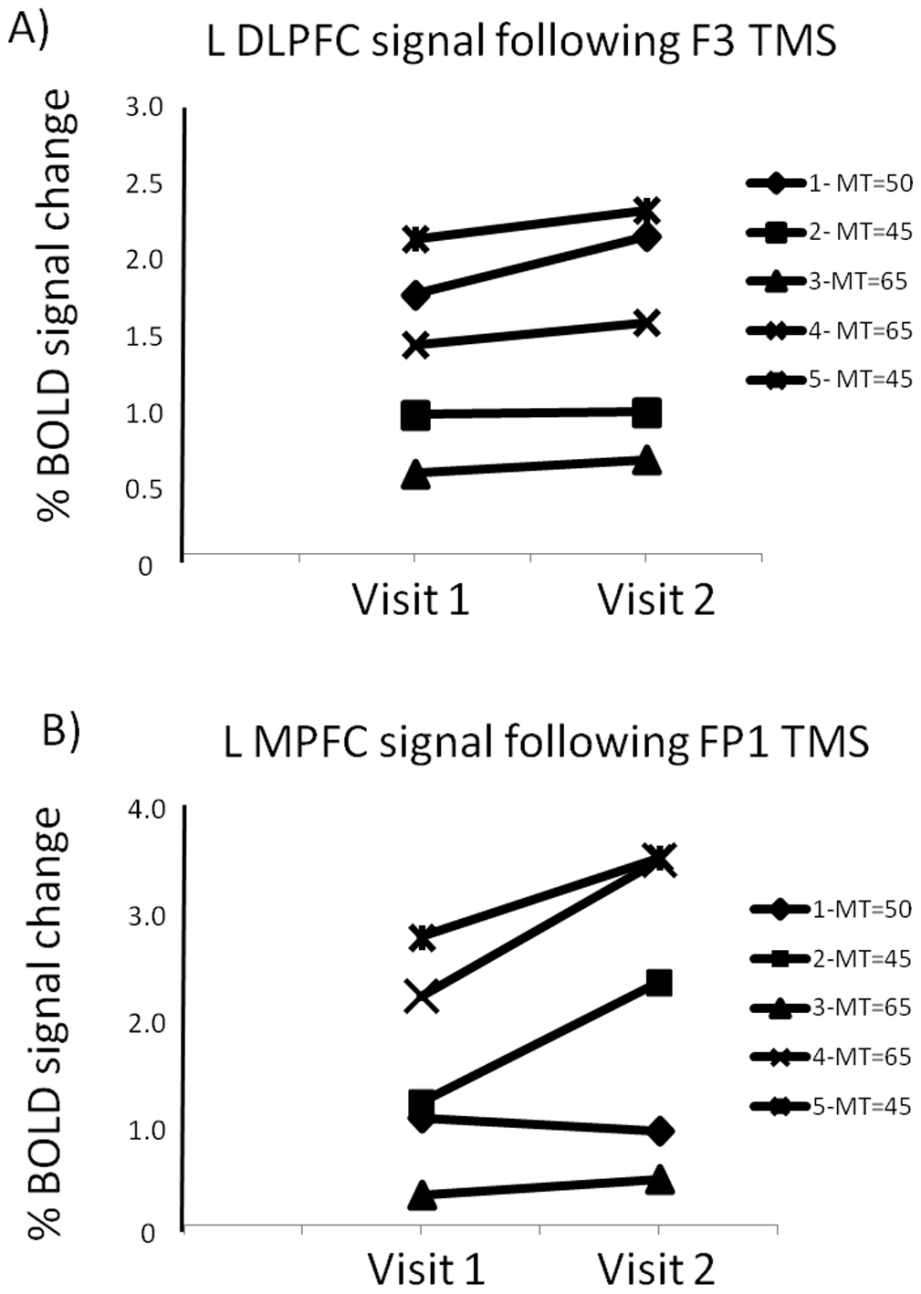
Percent signal change in the left DLPFC following stimulation at F3 (A) and the MPFC following stimulation at FP1 (B) for 5 participants who underwent the experiment on two visits at the same threshold of stimulation.

Within this small cohort of individuals, there was no significant difference in percent BOLD signal change between Visit 1 and Visit 2 at either the DLPFC (t = 1.36, p = 0.27) or the MPFC stimulation site (t = 1.85, p = 0.16). The average difference from Visit 1 to Visit 2 in the left DLPFC was 0.015% (sd: 0.015, range: 0.002-0.02). The average difference in the MPFC was 0.08% (sd: 0.06, range: 0.01–0.14). The ranked order of the brain response to stimulation on Visit 1 at both sites was identical at Visit 2. (**Figure 6**) The between-subject variability at each visit however was relatively high for both sites. The percent signal change in the left DLPFC following F3 stimulation varied from 0.06–0.24 (0.06–0.22 (Visit 1), 0.07–0.24 (Visit 2)) and the percent signal change in the MPFC following FP1 stimulation varied from 0.03–0.38 (0.03–0.3 (Visit 1), 0.05–0.38 (Visit 2)). Among these individuals there was no association between strength of stimulation (range of motor thresholds: 45–65% machine output) and the magnitude of BOLD signal change in the vicinity of the coil.

## Discussion

The current study demonstrates that, through the use of TMS-BOLD it is possible to differentially activate lateral and medial prefrontal cortical regions, as well as their respective subcortical targets including the dorsal and ventral caudate. In addition to supporting and extending prior investigations of DLPFC stimulation [32], [33], [34], this study is the first to demonstrate the feasibility of stimulating the MPFC and its striatal targets. The demonstration of a consistent, causal relationship between stimulation in the lateral and medial PFC and BOLD signal increase in subcortical brain regions which are differentially connected to those cortical targets suggests that the mesocortical and mesolimbic systems may be explored and targeted with TMS in future applications to neurologic and psychiatric disease.

### Stimulating Frontal-striatal Circuits with Prefrontal TMS

A number of prior studies have examined the association between a single-pulse of TMS over the motor cortex [24], [27], [29], [40], [43] or DLPFC [32], [33], [34] and a BOLD signal response in both the region being stimulated and its subcortical targets. This is the first study however, to investigate the effects of TMS on MPFC activity. Consistent with the pattern observed in these prior studies, single pulse TMS applied to the MPFC (FP1) at the motor threshold was associated with elevated activity in the cortex adjacent to the coil as well as in subcortical regions monosynaptically connected to it, including the caudate, putamen and thalamus, but not the hippocampus or visual cortex.

In addition to differentially modulating the medial and lateral prefrontal cortex via TMS, these data demonstrate that the subcortical targets of these areas may also be differentially modulated by prefrontal coil placement. The anatomical pathways underlying this cortical-subcortical connectivity have been firmly established in primate literature [1], [8], [44], [45]. For example, the orbitofrontal and ventromedial prefrontal (Brodmann Area10,11,12) project significantly more to the caudate than to the putamen, specifically the medial and ventral domains of the caudate [8], [10]. The DLPFC sends afferent projections to the putamen and the dorsal domains of the caudate, but, unlike the MPFC it also has afferent connections with the hippocampal and entorhinal corticies [46].

Although emerging data from diffusion tensor imaging and sophisticated retrograde and anterograde tracing has revealed that these ventromedial and dorsolateral frontostriatal circuits which Alexander and collegues first described (1986) are likely not entirely anatomically distinct and parallel, we do still believe that they are functionally complementary to one another. The projections between the DLPFC and hippocampus, for example, are thought to explain the strong role that the DLPFC has in working memory [5], [6], [7]. The projections between orbitofrontal, MPFC and striatal structures like nucleus accumbens are key element of the mesolimbic dopamine system that modulate reward processing [11]. Disruption in these circuits has been demonstrated in multiple psychiatric diseases including psychopathy [15], [16], eating disorders [17], and substance dependence [19], [20].

### Beyond the Striatum

In addition to the striatal regions and the thalamus which are all monosynaptically connected to the prefrontal cortex and critical elements of the frontostriatal loops, our regions of interest analysis also revealed significant activity in the hippocampus following lateral PFC stimulation. Although the hippocampus is frequently considered a node in the limbic network with strong connectivity to the amygdala, there are well established projections from the hippocampal formation to both the medial and to the lateral prefrontal cortex. In a study of 27 rhesus monkeys which used retrograde tracers to identify patterns of prefrontal cortex connectivity with the hippocampus, Barbas and Blatt (1995) found that the medial and the lateral PFC were associated with labeled neurons in the CA1 and subiculum regions of the hippocampal formation respectively [44]. The most abundant projections to the lateral prefrontal cortex were from the presubiculum. Although the connectivity between the hippocampal and parahippocampal areas and the frontal cortex are not entirely clear, these fibers likely travel through the cingulum bundle [45], [47], [48]. The cingulum bundle however forms a complex tract comprised of both short and long association fibers which have made it difficult untangle via current diffusion tractography methods. As tractography develops however we will likely have a greater understanding of some of the other cortical and subcortical areas which are activated by stimulation of the prefrontal cortex.

In addition to the hippocampus, post-hoc voxel based analyses of these data revealed several other cortical regions which had an elevated BOLD signal following TMS to the lateral prefrontal cortex (temporal cortex, insula) and the medial prefrontal cortex (orbitofrontal cortex, cingulate cortex). While anatomically these regions all are in relatively close proximity to the site of stimulation and are likely highly connected via short-fiber interneuron projections, it is difficult to determine whether these regions were a direct consequence of the TMS pulse or due to the nature of the stimulation site. The left F3 site for example is closer to the left ear and consequently the left superior temporal gyrus (which is the location of the primary auditory cortex) could merely be more active given the amplitude of the sound from the TMS pulse. Likewise, the participants frequently reported that while the medial prefrontal cortex was “not painful” it was “more startling” than the lateral PFC stimulation, an experience which may invoke the anterior cingulate cortex.

### Moving forward to the Clinic

These preliminary data demonstrate that it is possible to reliably activate cortical nodes differentially involved in executive and limbic processing. Additionally, as shown by others, prefrontal stimulation is further associated with BOLD activity in subcortical target regions [31], [32], [40], which provides us with a window for us to investigate frontostriatal connectivity. While this is interesting from a basic systems-level neurobiology perspective, it provides us with a new opportunity to investigate the relative disruption in these circuits in neurologic and psychiatric disease. In substance dependence for example, it is unclear whether relapse to drug use is associated with a heightened sensitivity of the ventromedial reward system to drug cues, or perhaps an attenuated or underdeveloped dorsalateral executive control system. If we were able to determine the relative contribution of theses circuits to things such as drug relapse, we could then use non-invasive brain stimulation techniques to either amplify or attenuate signal transduction in these frontal striatal systems, in conjunction with cognitive behavioral therapy. This line of reasoning and hope for the future extends beyond substance abuse however to many other diseases which have both an executive and limbic component including obsessive compulsive disorder, eating disorders, chronic pain, and post-traumatic stress disorder.

Given that this was a relatively small investigation designed to determine the efficacy and reliability of dissociating lateral from medial prefrontal circuits, a site which had not previously been investigated, there are several limitations which must be considered when interpreting the results. First, the sensitivity of the functional MRI protocol in this small, but pioneering experiment (relatively large voxel size, relatively few time points) did not provide us with adequate power to resolve subregions of interest within our larger regions (such as the caudate which receives projections from both dorsal motor and ventral limbic regions of the frontal cortex). These are certainly questions that should be addressed in future studies which extend these preliminary results to larger samples of healthy individuals as well as patients with specific psychiatric and neurologic symptoms. Another option to consider in future voxel-based analyses might be modeling these data with a non-parametric design. Additionally, as with other single-pulse TMS studies in the MR environment, there was no sham coil used for this study. It would be valuable to develop a robust “sham” stimulation for the MR environment because each TMS pulse has both a strong auditory and somatosensory component, that likely contributes to the BOLD signal results in various brain regions. For example, the thalamus was the brain region that exhibited the strongest percent signal change in the present experiment. The fact that the thalamus also acts as a relay for nearly all incoming sensory information in the brain as well as serving as a striatal-cortical relay, makes it difficult to evaluate the meaning of the BOLD signal response in this region. Finally, while this study was limited to healthy young adults with minimal age-related atrophy, future studies applying medial prefrontal TMS to patients and older adults should consider that the net amplitude of the TMS induced field is proportional to the distance between the skull and the cortex [49], and consequently the stimulation intensity may need to be higher to achieve an effect.

Considered together the results of the present study suggest that interleaved TMS/fMRI strategically placed over the prefrontal cortex can selectively and consistently activate both cortical and subcortical targets of the mesocortical and mesolimbic systems. As these lateral and medial prefrontal circuits likely interact to balance the cortical control of affective responses, differentiating mesocortical from mesolimbic activity is an area of great interest in human neuromaging [21], [22]. Interleaved TMS/BOLD imaging enables us to probe connectivity in these systems in a more causal and systematic manner, which is difficult in traditional functional connectivity analysis. Interleaved TMS/BOLD is an evolving tool for studying these circuits in health and disease and understanding network behavior. Given the critical roles that mesocortical and mesolimbic circuitry have on shaping behavior, the ability to specifically modulate these circuits with a combination of TMS and MRI, may illuminate neural circuit level dysfunction in patients with psychiatric diseases which was not previously possible.
